# Novel Insights into Surface Energies and Enhanced Gas-Sensing Capabilities of ZnGa_2_O_4_(111) via Ab Initio Studies

**DOI:** 10.3390/s25020548

**Published:** 2025-01-18

**Authors:** Cheng-Lung Yu, Yan-Cheng Lin, Sheng-Yuan Jhang, Jine-Du Fu, Yi-Chen Chen, Po-Liang Liu

**Affiliations:** 1 Graduate Institute of Precision Engineering, National Chung Hsing University, No. 145, Xingda Road, Taichung 40227, Taiwan; bowin@mxvantage.com (C.-L.Y.); joker212940@gmail.com (Y.-C.L.); z8908193@gmail.com (S.-Y.J.); hoshinocyc_jp@smail.nchu.edu.tw (Y.-C.C.); 2 Max Vantage WH Co., Ltd., 7F.-2, No. 936, Sec. 4, Taiwan Blvd., Taichung 40764, Taiwan; winia2585@gmail.com; 3 Department of Applied Materials and Optoelectronic Engineering, National Chi Nan University, No. 1, University Road, Puli Township, Nantou 54561, Taiwan

**Keywords:** Ab initio study, ZnGa_2_O_4_, work function change, gas sensor

## Abstract

This study investigates the surface energies and work function changes in ZnGa_2_O_4_(111) surfaces with different atomic terminations using ab initio density functional theory. It explores the interactions of gas molecules such as NO, NO_2_, and CH_3_COCH_3_ with Ga-terminated, O-terminated, and Ga-Zn-O-terminated surfaces. This study reveals previously unreported insights into how O-terminated surfaces exhibit enhanced reactivity with NO, resulting in significant work function changes of +6.42 eV. In contrast, Ga-terminated surfaces demonstrate novel interactions with oxidizing gases, particularly NO_2_, with a notable reduction in work function change of −1.63 eV, offering potential gas sensor technology advancements. Particularly notable is the Ga-Zn-O-terminated surface, which presents mixed characteristics influenced by the interplay of oxygen and metallic elements (gallium and zinc), leading to substantial work function changes of +4.97 eV for NO and +1.82 eV for NO_2_, thereby significantly enhancing sensitivity. This study unveils the previously unexplored roles of Ga-Zn-O-terminated ZnGa_2_O_4_ surfaces in optimizing semiconductor-based gas sensors, offering both oxidative and reductive potentials and making them versatile for diverse applications.

## 1. Introduction

Environmental sensing plays a crucial role in artificial intelligence (AI) systems, enabling real-time monitoring of pollutants and toxic substances in industrial and environmental settings. The integration of next-generation sensors with AI not only enhances the detection and response speed but also enables predictive analytics for pollution control. Among various materials, spinel-structured zinc gallate (ZnGa_2_O_4_ or ZGO) has gained attention for its high sensitivity and broad detection range, making it suitable for environmental monitoring and industrial safety applications. Spinel-structured ZnGa_2_O_4_ sensors have demonstrated wide-ranging applications in pollutant detection and water splitting [1,2,3,4]. Their high sensitivity and broad detection scope make them particularly effective in environmental monitoring. Enhancing the sensitivity and expanding the detection range of these sensors remain focal points of current research, with investigators exploring innovative material solutions and technological advancements to achieve more efficient environmental monitoring. J. C. Tung et al. reported on the adsorption of nitrogen dioxide (NO_2_), an oxidizing gas, and hydrogen sulfide (H_2_S), a reducing gas, on gallium-zinc-oxide-terminated ZnGa_2_O_4_(111) surfaces using the first-principles density functional theory (DFT) calculations [5]. The research reveals that the adsorption of a single NO_2_ molecule onto the gallium atom increases the work function change, reaching a maximum change of +0.97 eV, indicative of enhanced sensor sensitivity to oxidizing gases. In contrast, bonding an H_2_S molecule, which acts as a reducing agent, to the gallium atom decreases the work function significantly, with the most substantial change being −1.66 eV. These changes in electronic properties align with experimental observations from ZnGa_2_O_4_-based gas sensors, emphasizing the thin films’ potential to detect environmental pollutants. Furthermore, recent advancements in sensor technology have explored the doping of ZnGa_2_O_4_ with palladium (Pd) to enhance its gas-sensing capabilities [6]. Pd doping has significantly altered the work function change of ZnGa_2_O_4_, a critical factor in the sensor’s ability to detect and respond to gas molecules. Specifically, the maximum work function changes were +2.37 eV and −1.82 eV for two NO_2_ and H_2_S molecules adsorbed onto the surface Pd atom on ZnGa_2_O_4_(111), respectively, showing that Pd-decorated ZnGa_2_O_4_(111) is a suitable material in NO_2_/H_2_S gas detectors. Additionally, the adsorption characteristics of carbon monoxide (CO), a reducing gas, on both pristine and Pd-doped ZnGa_2_O_4_(111) surfaces reveal distinct changes in work function [7]. The work function change due to CO adsorption is measured at −0.55 eV on pristine surfaces. This change is significantly amplified by Pd doping, increasing to −0.79 eV, representing a 1.43-fold enhancement. Similarly, ZnGa_2_O_4_-based gas sensors were used to detect nitric oxide (NO) in the parts-per-billion range [8]. The surface of ZnGa_2_O_4_(111) attracts oxygen molecules from the air to form oxygen ions. These oxygen ions act as catalysts in reactions with NO molecules to form a NO_2_-like molecule on the ZnGa_2_O_4_(111) surface. Moreover, argon (Ar) plasma treatment can substantially alter the surface properties of ZnGa_2_O_4_(111), enhancing its applicability in NO gas sensing [9]. The sensor’s response of plasma-treated ZnGa_2_O_4_(111) to a 5 ppm NO gas concentration dramatically increased from 159.5% to 1276.1%, demonstrating a substantial improvement in performance with a detection limit of 2.4 ppb. Cross-selectivity tests among NO, carbon dioxide (CO_2_), carbon monoxide (CO), and sulfur dioxide (SO_2_) gases indicated that the ZnGa_2_O_4_ gas sensor possesses a high degree of selectivity toward NO gas. In the presence of an oxidizing gas on the *n*-type ZnGa_2_O_4_ semiconductor, electrons flow from the semiconductor to the oxidizing gas until Fermi level equilibrium is reached. This electron transfer results in surface energy band bending, where the difference between the work function and electron affinity of the target gases on ZnGa_2_O_4_(111) surfaces becomes crucial. For oxidizing gases, this band bending typically results in a positive change in the work function, reflecting an increase in the potential barrier that prevents electrons within the semiconductor from migrating to the surface. Consequently, this increases the surface resistance of the semiconductor. Conversely, the process is reversed when a reducing gas interacts with the *n*-type ZnGa_2_O_4_ semiconductor. Electrons flow from the reducing gas to the semiconductor until the Fermi level is balanced, forming an ohmic contact and decreasing the work function. When the work function decreases, the potential barrier is reduced as the surface becomes more favorable for accommodating or releasing electrons. With a lower potential barrier, free electrons within the semiconductor can migrate more easily to the surface, enhancing the surface charge density. This migration contributes to a reduction in surface resistance, as the increased availability of electrons on the surface improves its conductivity. Such interactions typically result in a negative change in the work function, effectively lowering the potential barrier and reducing the surface resistance. This dynamic emphasizes the crucial role of surface chemistry in semiconductor-based gas sensors. It highlights the differential responses dictated by the chemical nature of the interacting gases, i.e., oxidizing gases increasing and reducing gases decreasing the work function changes.

In developing efficient gas sensors, the properties of the sensor surface are critical determinants of performance. Particularly for semiconductor-based gas sensors such as those utilizing ZnGa_2_O_4_(111), the termination of the surface by different atomic layers can significantly influence gas adsorption capabilities and sensing efficiency. Recent advances in ammonia gas sensors have demonstrated the potential of nanostructured materials for enhanced sensing performance. For instance, the development of ammonia sensors based on Multi-Wall Carbon Nanofiber Field Effect Transistors (MWCNF-FETs) was reported [10]. By applying gate modulation, they achieved a 55% improvement in sensor response and enhanced reversibility under optimal gate biases. This approach highlights the importance of surface engineering and electrical modulation in improving the sensitivity and stability of gas sensors. Incorporating such novel techniques can lead to significant advancements in environmental monitoring and industrial applications. This study reinforces the relevance of investigating surface terminations and electronic properties of ZnGa_2_O_4_(111) surfaces, which are central to the current research. While previous studies have primarily focused on the gas-sensing properties of ZnGa_2_O_4_-based materials, a systematic comparison of different atomic terminations and their combined effects on gas adsorption behavior is still lacking. To address this gap, we systematically compare Ga-terminated, O-terminated, and Ga-Zn-O-terminated surfaces to evaluate their influence on surface energy and work function changes. We aim to provide deeper insights into these terminations that exhibit distinct behaviors regarding stability and sensitivity to gas molecules, highlighting their potential for various sensing applications. Additionally, we identify the optimal surface termination for gas sensing performance based on work function variations. By calculating the surface energies and evaluating the work function changes associated with different atomic terminations, we seek to understand how these surface characteristics affect the material’s adsorption properties. Additionally, changes in the work function will be evaluated to determine which surface termination offers the best performance in gas sensing applications. The outcomes of this research are expected to provide both theoretical and practical foundations for designing and developing gas sensors with enhanced sensitivity and selectivity, thereby significantly contributing to environmental monitoring and industrial safety applications.

## 2. Computational Methods

**Figure 1:**
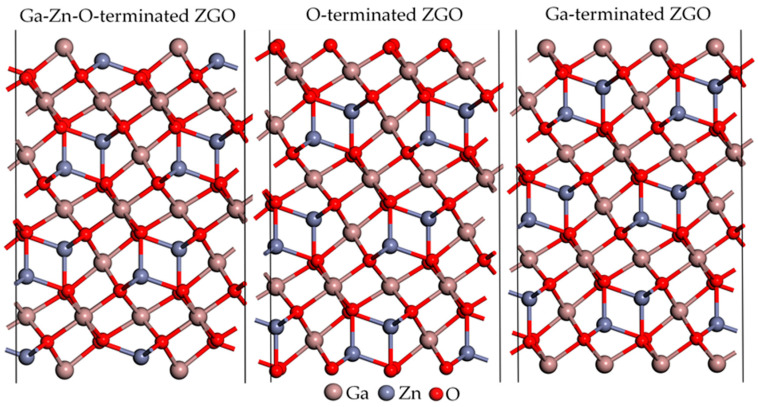
Schematic structures of Ga-Zn-O-terminated, O-terminated, and Ga-terminated ZnGa_2_O_4_(111) are considered in this study. Atoms are represented by spheres: Ga (brown, large), Zn (gray, medium-sized), and O (red, small).

For the theoretical investigation of the ZnGa_2_O_4_ surfaces, we utilized the Vienna ab initio simulation package (VASP) for all quantum mechanical simulations. VASP was employed in this study using the generalized gradient approximation (GGA) method coupled with the Perdew–Wang (PW91) functional for the exchange-correlation potential corrections [11,12,13,14]. The use of the GGA-PW91 functional is justified by its proven reliability in modeling the electronic structure and surface properties of transition metal oxides, as demonstrated in prior studies [1,5,6,7,8,9]. The bulk ZnGa_2_O_4_ was modeled in a cubic unit cell belonging to the *Fd*-3*m* space group, comprising 56 atoms: eight Zn, 16 Ga, and 32 O atoms, with lattice parameters *a*, *b*, and *c*, each measuring 8.33 Å. Plane-wave cutoff energies were initially set at 600 eV for the bulk calculations to ensure high precision in the total energy and electronic density distribution. The *k*-point sampling for the bulk phase calculations was performed using a 6 × 6 × 6 Monkhorst–Pack grid. The initial bulk model was segmented into three distinct surface terminations for the surface simulations: Ga-Zn-O-terminated, O-terminated, and Ga-terminated ZnGa_2_O_4_(111), depicted in Figure 1. These models aimed to elucidate the differential effects of atomic terminations on surface reactivity and properties. To ensure the accuracy of our surface calculations, we performed convergence tests to determine an optimal cutoff energy. While the initial bulk calculations used a cutoff energy of 600 eV for high precision, the significantly larger size of the surface models (172 to 184 atoms, including a vacuum layer of 44 Å) necessitated a reduction in the cutoff energy to maintain computational feasibility. The convergence tests showed that reducing the cutoff energy to 450 eV resulted in an energy deviation of less than 0.01 eV, confirming that the computational accuracy was maintained. Given the enlarged unit cell size, a 2 × 2 × 1 Gamma-centered grid was deemed sufficient. The geometrical optimizations of all models were conducted until the forces on each atom were minimized to less than 0.01 eV/Å, ensuring that the atomic positions represented stable configurations for subsequent electronic and adsorption property analyses.

The selected surface terminations in this study, i.e., Ga-terminated, O-terminated, and Ga-Zn-O-terminated ZnGa_2_O_4_(111) surfaces, are consistent with those commonly investigated in previous studies [1,5,6,7,8,9] on ZnGa_2_O_4_(111) surfaces, where Ga-Zn-O-terminated surface was primarily considered. This study further introduces a systematic analysis of Ga-terminated and O-terminated ZnGa_2_O_4_(111) surfaces, which have not been reported in previous literature. Regarding computational parameters, the cutoff energy of 450 eV and the 2 × 2 × 1 Gamma-centered *k*-point grid used in this study are consistent with previous works [1,5,6,7,8,9], ensuring reliable results while maintaining computational efficiency. Following previous studies on the ZnGa_2_O_4_(111) surface topology and active sites (Ref. [5]), we identified the Ga_3*c*_ and Zn_3*c*_ atoms as primary active sites for gas adsorption. Due to their lower coordination numbers and higher reactivity, these surface atoms are most likely to interact with incoming gas molecules. The NO adsorption on these active sites can significantly change the electronic structure and work function, which is crucial for enhancing gas sensor performance. Following previous studies on ZnGa_2_O_4_(111) surfaces (Ref. [8]), NO molecules react with surface oxygen atoms to form NO_2_-like structures, inducing significant electron transfer and dipole formation. This leads to notable changes in the work function and surface conductivity, which enhance the sensor’s performance. Previous first-principles studies have confirmed the thermodynamic favorability of this reaction, showing that the adsorption energy of NO on oxide-passivated ZnGa_2_O_4_ is sufficient to stabilize NO_2_-like adsorption complexes.

The chemical potential of ZnGa_2_O_4_ ($\mu_{{\mathrm{ZnGa}}_{2}O_{4}}^{\mathrm{bulk}}$) can be expressed as a function of the chemical potentials of its constituent elements, zinc ($\mu_{\mathrm{Zn}}$), gallium ($\mu_{\mathrm{Ga}}$), and oxygen ($\mu_{O}$). Under equilibrium conditions, the sum of the chemical potentials of the constituent elements, weighted by their stoichiometric coefficients in the compound, equals the chemical potential of ZnGa_2_O_4_. For the specific stoichiometry of ZnGa_2_O_4_, the relationship can be approximated by:

$$\mu_{\mathrm{Zn}}+2\mu_{\mathrm{Ga}}+4\mu_{O}=\mu_{{\mathrm{ZnGa}}_{2}O_{4}}^{\mathrm{bulk}}$$

Given that the stability of ZnGa_2_O_4_ is subject to the constraints imposed by the availability and chemical potentials of its constituent elements, the permissible range for each chemical potential is further constrained by the conditions of formation and stability of the bulk phases of each element as well as their common oxides. The allowable range for $\mu_{\mathrm{Zn}}$ and $\mu_{\mathrm{Ga}}$ is bound by their respective bulk metal and oxide formation potentials. Here, the permissible ranges for $\mu_{\mathrm{Zn}}$ and $\mu_{\mathrm{Ga}}$ are restricted within the thermodynamically allowed ranges as follows:

$$\mu_{\mathrm{Zn}}^{\mathrm{bulk}}+∆H^{f}\left[\mathrm{ZnO}\right]\;\leq \mu_{\mathrm{Zn}}\;\leq \;\mu_{\mathrm{Zn}}^{\mathrm{bulk}}$$

and

$$\mu_{\mathrm{Ga}}^{\mathrm{bulk}}+∆H^{f}\left[{\mathrm{Ga}}_{2}O_{3}\right]\;\leq \mu_{\mathrm{Ga}}\;\leq \;\mu_{\mathrm{Ga}}^{\mathrm{bulk}},$$

or simply

$$∆H^{f}\left[\mathrm{ZnO}\right]\;\leq \Delta \mu_{\mathrm{Zn}}\;\leq \;0$$

and

$$∆H^{f}\left[{\mathrm{Ga}}_{2}O_{3}\right]\;\leq \Delta \mu_{\mathrm{Ga}}\;\leq \;0,$$

where $∆H^{f}\left[\mathrm{ZnO}\right]$ and $∆H^{f}\left[{\mathrm{Ga}}_{2}O_{3}\right]$ are the formation enthalpies of ZnO and Ga_2_O_3_, respectively. The terms $\mu_{\mathrm{Zn}}$ and $\mu_{\mathrm{Ga}}$ represent the differences in the chemical potentials of zinc and gallium from their bulk states, calculated as $\mu_{\mathrm{Zn}}$ − $\mu_{\mathrm{Zn}}^{\mathrm{bulk}}$ and $\mu_{\mathrm{Ga}}$ − $\mu_{\mathrm{Ga}}^{\mathrm{bulk}}$, respectively. The chemical potentials of ZnO and Ga_2_O_3_, $\mu_{\mathrm{ZnO}}^{\mathrm{bulk}}$ and $\mu_{{\mathrm{Ga}}_{2}O_{3}}^{\mathrm{bulk}}$ were determined based on the stoichiometric relationships of their constituent elements using the following equations:

$${\mu_{\mathrm{Zn}}+\mu }_{O}=\mu_{\mathrm{ZnO}}^{\mathrm{bulk}}$$

and

$${2\mu_{\mathrm{Ga}}+3\mu }_{O}=\mu_{{\mathrm{Ga}}_{2}O_{3}}^{\mathrm{bulk}}.$$

The electronic configurations for valence electrons are O 2*s*^2^ 2*p*^4^, Zn 3*d*^10^ 4*s*^2^, and Ga 3*d*^10^ 4*s*^2^ 4*p*^1^. Our formation enthalpies of bulk ZnO and Ga_2_O_3_, $∆H^{f}\left[\mathrm{ZnO}\right]$ and $∆H^{f}\left[{\mathrm{Ga}}_{2}O_{3}\right]$, are determined from the total energies per atom of bulk ZnO (space group: 186 *P*63*mc*) and bulk Ga_2_O_3_ (space group: 12 *C*2/*m*), respectively, yielding $∆H^{f}\left[\mathrm{ZnO}\right]$ = −3.88 eV and $∆H^{f}\left[{\mathrm{Ga}}_{2}O_{3}\right]$ = −11.310 eV, in excellent agreement with other calculated value of $∆H^{f}\left[\mathrm{ZnO}\right]$ = −3.62 eV and $∆H^{f}\left[{\mathrm{Ga}}_{2}O_{3}\right]$ = −11.29 eV, respectively [15,16]. Specifically, the chemical potential ranges for Zn, Ga, and O were determined based on the formation enthalpies of ZnO and Ga_2_O_3_, with Ga-rich and O-rich conditions representing the extreme limits. Under Ga-rich conditions, the chemical potential of Ga approaches its bulk value, while under O-rich conditions, the chemical potential of O approaches the oxygen-rich limit. These ranges ensure the thermodynamic stability of ZnGa_2_O_4_ under different environmental conditions. The surface energy (σ) of the ZnGa_2_O_4_(111) was calculated using the formula:

$$\sigma =(E_{\mathrm{slab}}-\sum_{i}n_{i}\mu_{i})/2A,$$

where *E*_slab_ is the total energy of the vacuum-terminated slab, $n_{i}$ represents the number of atoms, $\mu_{i}$ denotes their chemical potentials, and *A* is the surface area. The work function (Φ_S_) was determined by the difference between the vacuum energy level (*E*_VAC_) and the Fermi energy (*E_F_*) of the ZnGa_2_O_4_(111) slab:

$$\Phi_{S}=E_{\mathrm{VAC}}-E_{F}.$$

The relationship between work function changes and gas sensor performance can be quantitatively expressed using the ratio of the sensor’s resistance in the presence of the target gas (Rg) to its resistance in air (Ra) [5]. The work function difference (∆*Φ*) is given by the equation ∆*Φ* = ∆*X* + *kT*ln(Rg/Ra), where ∆*X* represents the change in electron affinity, and *kT* is the product of the Boltzmann constant and temperature. When an oxidizing gas is adsorbed on the ZnGa_2_O_4_(111) surface, upward band bending occurs, leading to a depletion of free charge carriers and an increase in resistance, corresponding to a positive work function change. Conversely, exposure to reducing gases causes downward band bending, increasing the density of free charge carriers, reducing resistance, and resulting in a negative work function change. Therefore, the distinct work function changes for different gas types are critical in determining the sensor’s sensitivity and selectivity.

## 3. Results and Discussion

**Figure 2:**
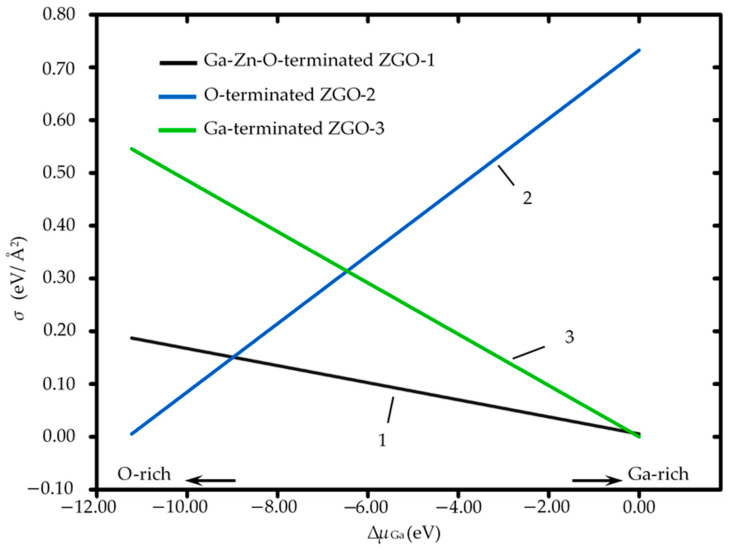
Comparison of Ga-Zn-O-terminated, O-terminated, and Ga-terminated ZnGa_2_O_4_(111) surface energies as a function of $\mu_{\mathrm{Ga}}$, where $\mu_{\mathrm{Ga}}$ represents the chemical potential difference relative to metallic gallium (Ga bulk) and is defined as $\mu_{\mathrm{Ga}}$ = $\mu_{\mathrm{Ga}}$ − $\mu_{\mathrm{Ga}}^{\mathrm{bulk}}$.

This study evaluated the surface energy and stability of ZnGa_2_O_4_(111) under different surface termination conditions, namely Ga-Zn-O-terminated, O-terminated, and Ga-terminated ZnGa_2_O_4_(111), in a vacuum environment. As shown in Figure 2, Ga-Zn-O-terminated ZnGa_2_O_4_(111) exhibits greater stability over a wide range of allowed chemical potentials compared to the O-terminated surface. The stability of the different surface terminations is assessed as a function of the chemical potential difference of gallium ($\mu_{\mathrm{Ga}}$), which has been previously defined in the Computational Methods section. This analysis provides insights into the relative stability of surface terminations under Ga-rich and Ga-poor conditions. The observation is that the Ga-terminated ZnGa_2_O_4_(111) exhibits the lowest surface energy of 0.0464 eV/Å^2^ only under gallium-rich conditions. At the same time, the Ga-Zn-O-terminated ZnGa_2_O_4_(111) generally maintains the lowest surface energy across a broader range of chemical potentials. This broader stability makes it more versatile for practical applications where environmental conditions cannot be strictly controlled. Therefore, Ga-Zn-O-terminated ZnGa_2_O_4_(111) has significant implications for these materials’ practical application and stability in various environments.

In gas sensing technologies, the surface energy of a material critically influences its reactivity and sensitivity. Surface energy, defined as the energy required to form a material’s surface, is directly correlated with the chemical activity of that surface. For Ga-Zn-O-terminated ZnGa_2_O_4_(111), the relatively low surface energy typically indicates a less active surface due to the lower energy required for its formation. This characteristic could reduce the material’s gas adsorption capabilities, limiting the sensor’s initial efficiency in capturing gas molecules. Conversely, the Ga-terminated ZnGa_2_O_4_(111) surfaces might show decreased chemical activity, but this low activity is restricted to a narrower range of chemical potentials. The Ga-terminated ZnGa_2_O_4_(111) displays a higher surface energy in an atmospheric environment, suggesting that high activity facilitates enhanced adsorption of gas molecules, improving the sensor’s reactivity and sensitivity. Given these properties, it is perhaps unsurprising that the Ga surface atoms on the Ga-Zn-O-terminated ZnGa_2_O_4_(111) could serve as critical sensitivity sites in ZnGa_2_O_4_-based gas sensors. Past studies have shown that Ga surface atoms can be key sensitive sites in ZnGa_2_O_4_-based gas sensors [5,6,7,8,9].

**Figure 3:**
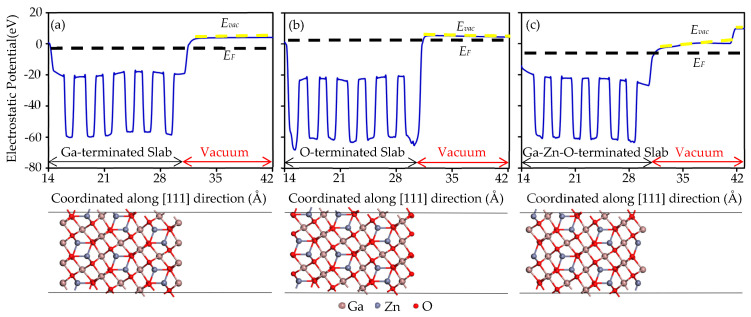
Planar average (solid line) and vacuum level E_VAC_ and Fermi level E_F_ (dashed lines) of electrostatic potential near (**a**) Ga-terminated, (**b**) O-terminated, and (**c**) Ga-Zn-O-terminated ZnGa_2_O_4_(111) surface computed within DFT-GGA functional. Atoms are represented by spheres: Ga (brown, large), Zn (gray, medium-sized), and O (red, small).

Figure 3 shows surface potential and work function analysis of Ga-Zn-O-terminated, O-terminated, and Ga-terminated ZnGa_2_O_4_(111). The work function of a material is defined as the minimum energy required to remove an electron from the surface of a solid and transport it to a point where the forces exerted by the material no longer influence it. The magnitude of the work function directly impacts how easily electrons can escape from the material’s surface. In Figure 3a, the electrostatic potential diagram for the Ga-terminated ZnGa_2_O_4_(111) surface reveals a vacuum level at 3.91 eV and a Fermi level at −2.45 eV, resulting in a work function of 6.36 eV. This is the highest work function among the three terminations examined, indicating the strongest electron repulsion by the Ga-terminated surface. Such a high work function implies a high resistance to chemical reactions, which could translate into enhanced stability and inertness in reactive environments. In Figure 3b, the electrostatic potential diagram for the O-terminated ZnGa_2_O_4_(111) surface illustrates a vacuum level at 4.35 eV and a Fermi level at 3.87 eV. This configuration yields a work function of 0.48 eV. The relatively low work function suggests a weaker electron repulsion by the O-terminated surface, which could be advantageous for the adsorption of gas molecules. This characteristic might enhance surface interactions with molecules that are electron donors, potentially increasing the sensitivity of sensors based on this material. Furthermore, materials with a low work function allow electrons to escape more easily, as less energy is required to overcome the energy barrier. This trait suggests that electrons are more loosely held, which may lead to higher chemical activity.

For the Ga-Zn-O-terminated ZnGa_2_O_4_(111), the electrostatic potential diagram indicates a vacuum level at 0.31 eV and a Fermi level at −3.39 eV, leading to a work function of 3.70 eV. This surface incorporates not only gallium (Ga) atoms but also zinc (Zn) and oxygen (O) atoms, forming a composite structure. This surface structure modifies the local electronic environment around the Ga atoms. Specifically, on the Ga-Zn-O-terminated surface, the proximity of Zn and O atoms influences the electronic effects around the Ga atoms. The high electronegativity of oxygen atoms can increase the electron density in the neighboring regions, making Ga atoms in this area more likely to form stable interactions with gas molecules that have strong electron affinities. Consequently, Ga atoms on such composite surfaces might exhibit enhanced chemical activity compared to those on a purely Ga-terminated surface. Additionally, the differences in electronegativity among the elements on the surface can create microelectronic fields on the Ga-Zn-O-terminated surface. These fields help attract and direct gas molecules to the Ga atoms, thereby enhancing the sensing response to specific gases. Thus, the Ga-Zn-O-terminated surface structure provides important physical and chemical mechanisms in designing and optimizing gas sensing materials, significantly enhancing the material’s sensing performance and application potential.

Table 1 meticulously details the work function changes of NO, NO_2_, and CH_3_COCH_3_ gas molecules across three distinct surface terminations of Ga-terminated, O-terminated, and Ga-Zn-O-terminated ZnGa_2_O_4_(111), providing a direct quantification of the adsorption effects exhibited by the various surfaces. Here, spin-polarized calculations were not included in this study. Preliminary tests on the Ga-terminated ZnGa_2_O_4_(111) surface without adsorbed molecules indicated that the inclusion of spin polarization resulted in a negligible change in the work function, increasing from 6.36 eV to 6.41 eV. This minimal difference suggests that spin polarization effects are unlikely to significantly impact the computed surface properties or alter the main conclusions of this study. The adsorption of NO molecules on the O-terminated surface results in a work function increase of 6.42 eV, indicating an exceptionally high adsorption capacity and sensitivity to NO molecules. The Ga-Zn-O-terminated surface shows a work function increase of 4.97 eV, suggesting robust adsorption capability for NO. In comparison, the Ga-terminated surface shows a more modest increase of 1.42 eV, indicating weaker adsorption capacity for NO. For NO_2_ molecules, the Ga-Zn-O-terminated surface displays the highest increase in work function by 1.82 eV post-adsorption, showcasing substantial adsorption. The O-terminated surface experiences a work function change of 1.77 eV due to NO_2_ adsorption, comparable to the Ga-Zn-O-terminated surface. The Ga-terminated surface exhibits a unique negative change of −1.63 eV, suggesting a reduction reaction that may adversely impact the surface’s electronic properties due to the reductive nature of NO_2_. When NO_2_ molecules are adsorbed onto such surfaces, they may engage in significant oxidative reactions with the surface oxygen atoms, capturing electrons and consequently leading to a positive shift in the work function. In contrast, gallium exhibits lower electronegativity compared to oxygen, which may result in less effective electron attraction when NO_2_ is adsorbed, possibly triggering a reduction reaction by releasing electrons to the gallium-terminated surface, thus decreasing the work function and causing a negative shift in the work function change. The formation of electric fields on these surfaces influences electron dynamics; the field on the O-terminated surface tends to attract NO_2_ electrons, whereas on the Ga-terminated surface, it may facilitate electron release. These phenomena collectively contribute to the observed variances in work function changes, even for the same gas molecule on different surface terminations of the same semiconductor material. The content related to adsorption energies from previous first-principles calculations can be found in the Supplementary Information: Analysis of Adsorption Energies. Specifically, the initial configurations of NO molecules on the ZnGa_2_O_4_(111) surface, covering vertical and horizontal adsorption models, and the optimized adsorption structures are described in detail. These details are presented in Figures S1 and S2 for visual clarity and further analysis.

Our computational results are consistent with experimental observations regarding work function changes and enhanced sensor performance [9]. First-principles calculations indicate a +0.54 eV work function change for the O-terminated ZnGa_2_O_4_(111) surface upon NO adsorption, which is approximately 2.3 times higher than that of the Ga-Zn-O-terminated surface. This aligns with the experimental observation of an 8-fold increase in ZnGa_2_O_4_-based NO gas sensor response after Ar plasma treatment. Furthermore, the computational model suggests that oxygen vacancies play a critical role in NO adsorption, which agrees well with X-ray photoelectron spectroscopy (XPS) measurements showing a significant increase in oxygen vacancy concentration after 10 min of Ar plasma treatment. These findings collectively confirm the computational predictions’ validity and relevance to real-world sensor performance. The Ga-Zn-O-terminated ZnGa_2_O_4_(111) surface shows high thermodynamic stability, which is crucial for maintaining a stable surface structure under varying gas environments in practical sensor applications. Previous studies have demonstrated that ZnGa_2_O_4_-based gas sensors can effectively detect NO gas, with surface stability and NO adsorption behavior being critical factors influencing sensor performance [1,8,9]. Our computational results indicate that the Ga-Zn-O termination offers a balanced interaction with oxidizing and reducing gases, enhancing the sensitivity and selectivity of ZnGa_2_O_4_-based sensors. These characteristics suggest that Ga-Zn-O-terminated ZnGa_2_O_4_ surfaces can contribute to the development of high-performance gas sensors with improved long-term stability. Understanding these nuances is crucial for designing semiconductor sensors tailored for specific gas detection applications, highlighting the intersection of material science and sensor technology.

Additionally, the analysis of CH_3_COCH_3_ adsorption reveals varying effects on the work function depending on the surface termination of ZnGa_2_O_4_(111). For the O-terminated surface, CH_3_COCH_3_ adsorption results in a positive work function change of 0.82 eV, indicating an oxidation process likely facilitated by the high electronegativity of oxygen atoms. This suggests that the O-terminated surface can effectively capture electrons from the acetone molecule, enhancing the surface’s oxidation capacity. In contrast, the Ga-Zn-O-terminated surface shows a negative change in work function of −1.26 eV upon adsorption of CH_3_COCH_3_. This indicates a reduction reaction, possibly due to the unique interaction between the surface atoms and the molecular structure of acetone, which may facilitate a transfer of electrons to the surface rather than capturing them, leading to an increase in surface charge density. On the Ga-terminated surface, the slight negative change of −0.48 eV in work function following CH_3_COCH_3_ adsorption also suggests a reduction effect but to a lesser extent. This might be attributed to the lower electronegativity of gallium than oxygen, making it less effective at attracting electrons from the acetone molecules. These observations imply that the termination layers’ surface chemistry and electronic properties are crucial in determining the interaction dynamics with CH_3_COCH_3_ molecules. The ability of oxygen to attract and stabilize additional electrons seems to enhance the oxidation reactions on O-terminated surfaces. In contrast, the presence of gallium might lead to a more reductive environment on Ga-terminated and Ga-Zn-O-terminated surfaces. Understanding these variations is essential for optimizing surface designs in applications such as catalysis and gas sensing, where specific surface reactions are desirable.

**Figure 4:**
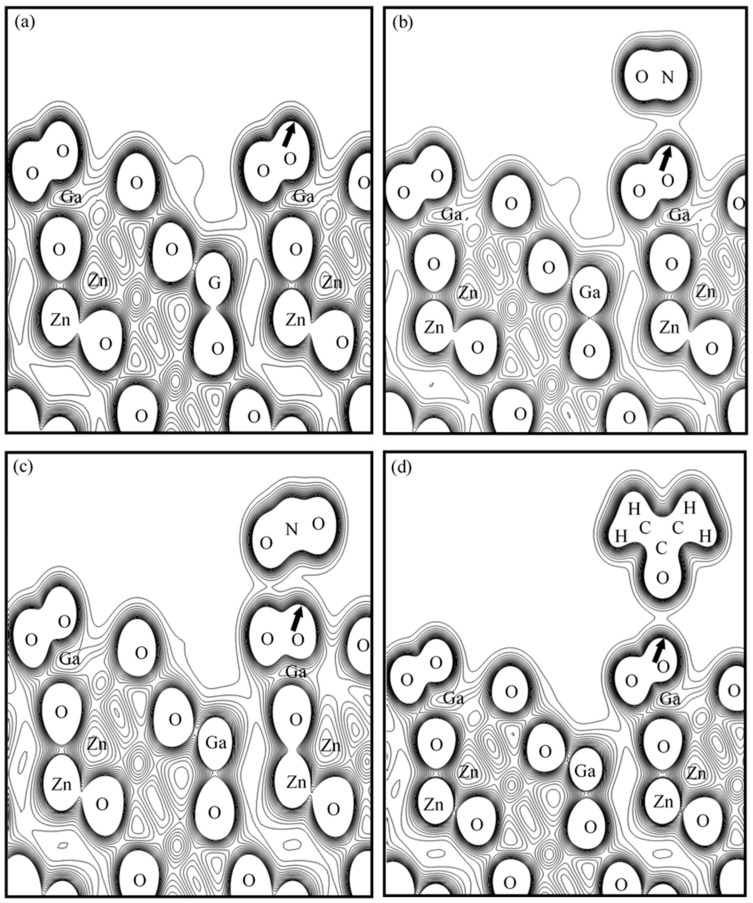
The charge density maps of O-terminated ZnGa_2_O_4_(111) before and after gas adsorption: (**a**) O-terminated ZnGa_2_O_4_(111) surface with an initial electron density of 0.432 electrons/Å^3^, featuring 15 contour levels with an interval of 0.027 electrons/Å^3^; (**b**) NO adsorption resulting in an electron density of 0.448 electrons/Å^3^, with 15 contour levels at an interval of 0.028 electrons/Å^3^; (**c**) NO_2_ adsorption resulting in an electron density of 0.435 electrons/Å^3^, featuring 14 contour levels with an interval of 0.029 electrons/Å^3^; and (**d**) CH_3_COCH_3_ adsorption resulting in an electron density of 0.480 electrons/Å^3^, with 15 contour levels at an interval of 0.030 electrons/Å^3^. The changes in electron density indicate varying degrees of charge transfer during the adsorption process, with NO and CH_3_COCH_3_ showing significant interactions compared to NO_2_. The arrows indicate the position on the ZnGa_2_O_4_(111) surface where the electron density is highest, representing regions of strong electronic interaction and potential adsorption activity.

To illustrate the relationship between electron density variations and adsorption behavior, we selected the O-terminated ZnGa_2_O_4_(111) surface for detailed analysis, as shown in Figure 4. This surface represents oxygen-passivated conditions typically observed in atmospheric environments, making it highly relevant for practical gas sensing applications. The initial electron density of the O-terminated surface is 0.432 electrons/Å^3^. The electron density increases to 0.448 electrons/Å^3^ upon NO adsorption, indicating significant charge transfer and strong interaction between NO molecules and the surface. NO_2_ adsorption results in a minor increase in electron density to 0.435 electrons/Å^3^, reflecting weaker interactions. The significant increase in electron density upon NO adsorption indicates substantial charge transfer during the adsorption process, leading to an increase in the surface positive potential. This observation is consistent with the notable work function change of +6.42 eV shown in Table 1. In contrast, the slight increase in electron density after NO_2_ adsorption suggests weaker charge transfer, resulting in a smaller work function change of +1.77 eV. For CH_3_COCH_3_ adsorption, the electron density increases significantly to 0.480 electrons/Å^3^, despite the larger size of the molecule. Since CH_3_COCH_3_ is a large organic molecule consisting of 10 atoms, its adsorption process involves a larger molecular structure compared to smaller molecules like NO and NO_2_. As a result, the interaction primarily occurs at specific high-activity sites on the surface, leading to localized electron transfer and a smaller overall work function change. These results highlight the critical role of surface electron density variations in influencing gas sensing performance. The significant work function change observed upon NO adsorption suggests enhanced sensitivity to oxidizing gases, making O-terminated ZnGa_2_O_4_(111) a promising candidate for detecting NO, in full agreement with the previous experimental studies [1,8,9]. In contrast, the smaller work function change for NO_2_ indicates its relatively weaker interaction, which may affect selectivity in mixed gas environments. The adsorption of CH_3_COCH_3_, despite its large molecular size, demonstrates the capability of the O-terminated surface to interact with complex organic molecules. This behavior could be advantageous in detecting volatile organic compounds (VOCs), suggesting potential applications in environmental monitoring and industrial safety. However, the localized nature of the electron transfer might lead to a lower sensitivity compared to smaller gas molecules. In addition to sensitivity, the selectivity of different ZnGa_2_O_4_(111) terminations for specific gases was also analyzed. The computational results indicate that the O-terminated surface demonstrates good selectivity for NO and NO_2_, as evidenced by significant work function changes (+6.42 eV for NO and +1.77 eV for NO_2_) and electron density variations upon adsorption. This suggests that the specific interactions between the O-terminated surface and these oxidizing gases are key in determining selectivity. Moreover, experimental studies by Refs. [1,9] confirmed the high selectivity of O-terminated ZnGa_2_O_4_ sensors for NO detection, further supporting our findings.

## 4. Conclusions

The research highlights how surface termination on ZnGa_2_O_4_(111) is pivotal in determining the adsorption characteristics and electronic interactions with different gas molecules. Ga-Zn-O-terminated surfaces generally provide the best overall performance for gas sensing applications. They show a versatile response to oxidizing and reducing gases due to their moderate work function changes and stable chemical interaction patterns. These findings offer valuable insights into the design and development of advanced semiconductor gas sensors, emphasizing the necessity of tailoring surface properties to enhance sensitivity and selectivity for environmental monitoring and industrial safety applications. Future studies will optimize the dopant concentrations and explore other surface modifications to enhance sensor performance across a broader range of gas species.

## Figures and Tables

**Table 1:** Calculated work function changes (Δ*Φ*) resulting from the adsorption of gas molecules NO, NO_2_, and CH_3_COCH_3_ on various terminated surfaces of ZnGa_2_O_4_(111): Ga-terminated O-terminated, and Ga-Zn-O-terminated. The plot includes values for the vacuum energy level (*E*_VAC_), the Fermi energy level (*E*_F_), the work function (*Φ_S_*) of the pristine ZnGa_2_O_4_(111) surfaces, and the work function after gas adsorption (*Φ_S,gas_*). Δ*Φ* represents the difference in work function before and after gas adsorption, illustrating the impact of molecular interaction on the surface electronic properties. All energies are presented in eV.

Models	*E*_VAC_ (eV)	*E*_F_ (eV)	Φ*_S,gas_* (eV)	Φ*_S_* (eV)	ΔΦ (eV)
Ga-terminated ZnGa_2_O_4_(111)	3.91	−2.45	-	6.36	-
O-terminated ZnGa_2_O_4_(111)	4.35	3.87	-	0.48	-
Zn-Ga-O-terminated ZnGa_2_O_4_(111)	0.31	−3.39	-	3.70	-
Ga-NO ZnGa_2_O_4_(111)	6.25	−1.53	7.78	-	1.42
O-NO ZnGa_2_O_4_(111)	4.54	−2.36	6.9	-	6.42
Zn-Ga-O-NO ZnGa_2_O_4_(111)	7.63	−1.04	8.67	-	4.97
Ga-NO_2_ ZnGa_2_O_4_(111)	3.02	−1.71	4.73	-	−1.63
O-NO_2_ ZnGa_2_O_4_(111)	7.46	5.21	2.25	-	1.77
Zn-Ga-O-NO_2_ ZnGa_2_O_4_(111)	3.33	−2.19	5.52	-	1.82
Ga-CH_3_COCH_3_ ZnGa_2_O_4_(111)	4.00	−1.88	5.88	-	−0.48
O-CH_3_COCH_3_ ZnGa_2_O_4_(111)	−2.22	−3.52	1.30	-	0.82
Zn-Ga-O-CH_3_COCH_3_ ZnGa_2_O_4_(111)	0.84	−1.60	2.44	-	−1.26

## Data Availability

The data presented in this study are available on request from the corresponding author.

## Supplementary Materials

The following supporting information can be downloaded at: https://www.mdpi.com/article/10.3390/s25020548/s1.
