# Supplementary material for: Novel Insights into Surface Energies and Enhanced Gas-Sensing Capabilities of ZnGa2O4(111) via Ab Initio Studies
Source: Sensors (Basel). 2025 Jan 18;25(2):548. doi: 10.3390/s25020548 (PMC11768928; doi:10.3390/s25020548)
Supplement: Supplementary file 1 [file sensors-25-00548-s001.zip › sensors-3360678-supplementary.pdf]

Supplementary Information Analysis of Adsorption Energies

Title: Novel Insights into Surface Energies and Enhanced Gas-Sensing Capabilities of $ZnGa_2O_4(111)$ via *Ab Initio* Studies

Authors: Cheng-Lung Yu, Yan-Cheng Lin, Sheng-Yuan Jhang, Jine-Du Fu, Yi-Chen Chen, Po-Liang Liu

In this supplementary information, we present a detailed description and analysis of the adsorption energies for NO molecules on Ga-Zn-O-terminated $ZnGa_2O_4(111)$ surfaces based on the constructed models from previous first-principles calculations as shown in Figures S1 and S2.

1. Computational Settings

The computational parameters employed here were carefully chosen to ensure accurate modeling of the geometric optimization and adsorption energy calculations for NO molecules on Ga-Zn-O-terminated $ZnGa_2O_4(111)$ surfaces. The exchange-correlation functional was described using the Generalized Gradient Approximation (GGA) with the Perdew-Wang 91 (PW91) method for correction. A cut-off energy of 600 eV was consistently applied to both $ZnGa_2O_4(111)$ surfaces and NO molecules to achieve high precision in energy calculations. The *k*-points grid was selected based on the model type: a 6 × 6 × 6 Monkhorst-Pack grid for the $ZnGa_2O_4$ bulk model, a 3 × 3 × 1 Gamma-centered grid for the Ga-Zn-O-terminated $ZnGa_2O_4(111)$ surface model, and a 1 × 1 × 1 Monkhorst-Pack grid for the isolated NO molecule. The surface models was constructed with 112 atoms, comprising 16 Zn atoms, 32 Ga atoms, and 64 O atoms, and included a vacuum layer thickness of 40 Å to eliminate interactions between periodic images. The initial placement of NO molecules at various adsorption sites was determined using the sum of the van der Waals radii of the interacting atoms, with the following values: 1.55 Å for the N atom, 1.52 Å for the O atom, 1.39 Å for the Zn atom, and 1.87 Å for the Ga atom. These computational settings were carefully designed to balance accuracy with computational efficiency, ensuring reliable predictions of the interaction behavior between NO molecules and the Ga-Zn-O-terminated $ZnGa_2O_4(111)$ surface.

2. Description of Models and Adsorption Energies

The adsorption models for NO molecules on $ZnGa_2O_4(111)$ surfaces were constructed and analyzed based on two primary configurations: vertical and horizontal orientations. In the vertical adsorption models (NO-N1 to NO-O4) as shown in Figure S1, the NO molecule was positioned perpendicularly to

the Ga-Zn-O-terminated $ZnGa_2O_4(111)$ surface at various active sites, including Ga_{3c}, Zn_{3c}, O_{3c}, and O_{4c}. Specifically, in models NO-N1 to NO-N4, the N atom of the NO molecule was directed toward the surface atoms at the respective sites, while in models NO-O1 to NO-O4, the O atom faced the surface. Following geometric optimization, the adsorption energies (ΔE) for the vertical models ranged from −1.45 eV to −0.01 eV, reflecting significant variations in interaction strength depending on the adsorption site. Among these, models NO-N1 and NO-N4 exhibited the highest adsorption energies of −1.45 eV and −1.41 eV, respectively, indicating strong chemical bonding

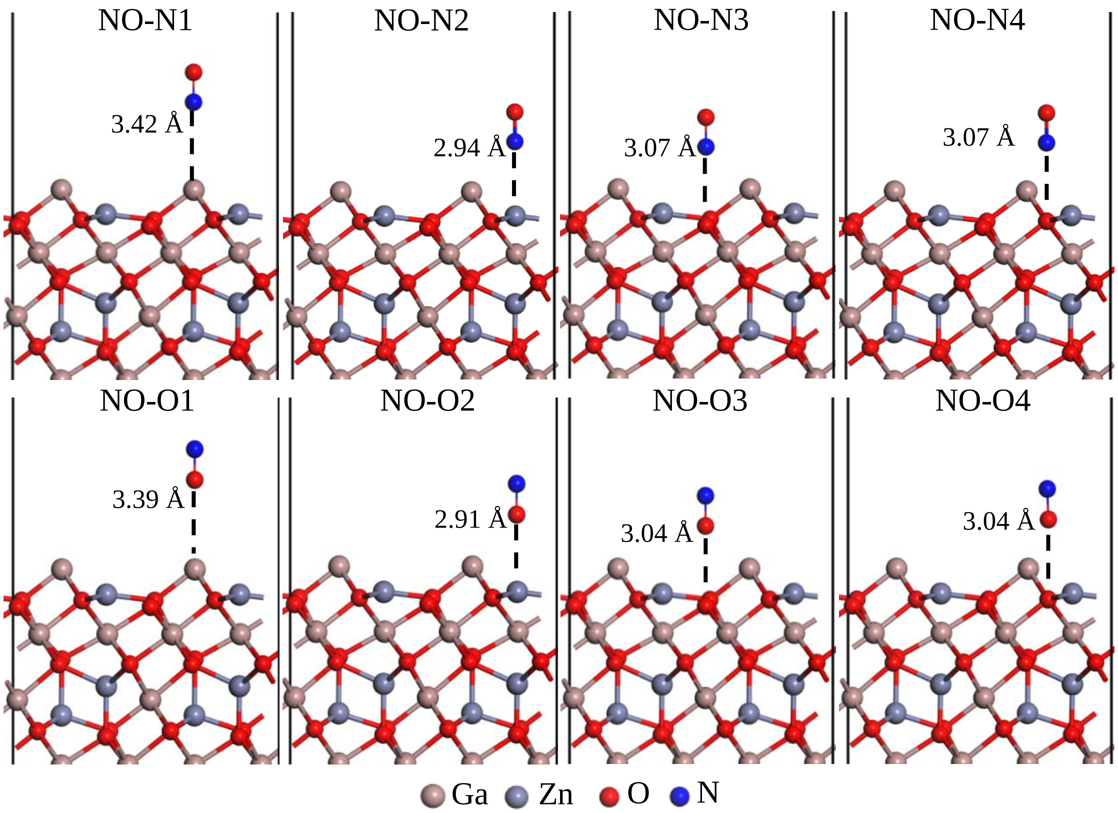

Figure S1 Initial configurations of NO molecules vertically adsorbed on the $ZnGa_2O_4(111)$ surface before geometric optimization. In models NO-N1 to NO-N4, the NO molecules are positioned with the nitrogen atom facing downward toward the preferred adsorption sites of Ga_{3c}, Zn_{3c}, O_{3c}, and O_{4c} positions, while in models NO-O1 to NO-O4, the oxygen atom faces downward toward the corresponding adsorption sites of Ga_{3c}, Zn_{3c}, O_{3c}, and O_{4c} positions on the $ZnGa_2O_4(111)$ surface. Ga atoms are depicted in brown, Zn atoms in gray, O atoms in red, and N atoms in dark blue.

with Ga atoms. In contrast, models NO-O1 to NO-O4 showed weaker adsorption, with energy values ranging from −0.48 eV to −0.01 eV, suggesting that direct interaction of the O atom with the surface atoms resulted in less favorable adsorption. The horizontal adsorption models (NO-N5 to NO-O8) as shown in Figure S2 involved placing the NO molecule parallel to the Ga-Zn-O-terminated $ZnGa_2O_4$(111) surface at the same active sites. In models NO-N5 to NO-N8, the N atom was positioned closer to the surface at the Ga_{3c}, Zn_{3c}, O_{3c}, and O_{4c} sites, while in models NO-O5 to NO-O8, the O atom was placed closer to these sites. After optimization, the adsorption energies ranged from −1.49 eV to −0.01 eV, closely matching the range observed in the vertical models, albeit with slightly lower values in certain configurations. Notably, models NO-N5 and NO-N9 displayed the strongest adsorption, with energies of −1.48 eV and −1.45 eV, respectively, indicating robust and stable adsorption complexes. This comprehensive analysis underscores the influence of adsorption orientation and surface site reactivity on the overall adsorption strength of NO molecules on $ZnGa_2O_4$(111) surfaces.

3. Analysis of Adsorption Energy Trends

Strong adsorption was observed at Ga_{3c} and Zn_{3c} sites, where the highest adsorption energies were recorded due to significant charge transfer. This behavior aligns with the known high reactivity of Ga and Zn atoms in the $ZnGa_2O_4$ lattice. In contrast, weaker adsorption occurred at O_{3c} and O_{4c} sites, where lower adsorption energies were observed. These results suggest that oxygen sites on the $ZnGa_2O_4$(111) surface are less reactive toward NO molecules, resulting in weaker binding and minimal energy changes. A comparative analysis of vertical and horizontal adsorption models revealed that horizontal adsorption generally led to slightly stronger binding, which can be attributed to the increased interaction surface area in horizontal configurations, especially when the N atom was positioned near reactive surface sites.

4. Significance of Adsorption Energy Variations

The variations in adsorption energies highlight the critical role of surface site reactivity and NO orientation in gas sensing performance. Specifically, high adsorption energies ($\Delta E < -1.4$ eV) indicate strong chemical adsorption, which is advantageous for achieving high sensitivity in gas sensors. In contrast, moderate to low adsorption energies ($\Delta E > -0.5$ eV) suggest weaker interactions, which are beneficial for reversible adsorption, a key feature for sensor recovery. These findings demonstrate that the $ZnGa_2O_4$(111) surface, particularly at Ga_{3c} and Zn_{3c} sites, offers strong and stable adsorption

properties, making it a promising material for NO gas sensing applications.

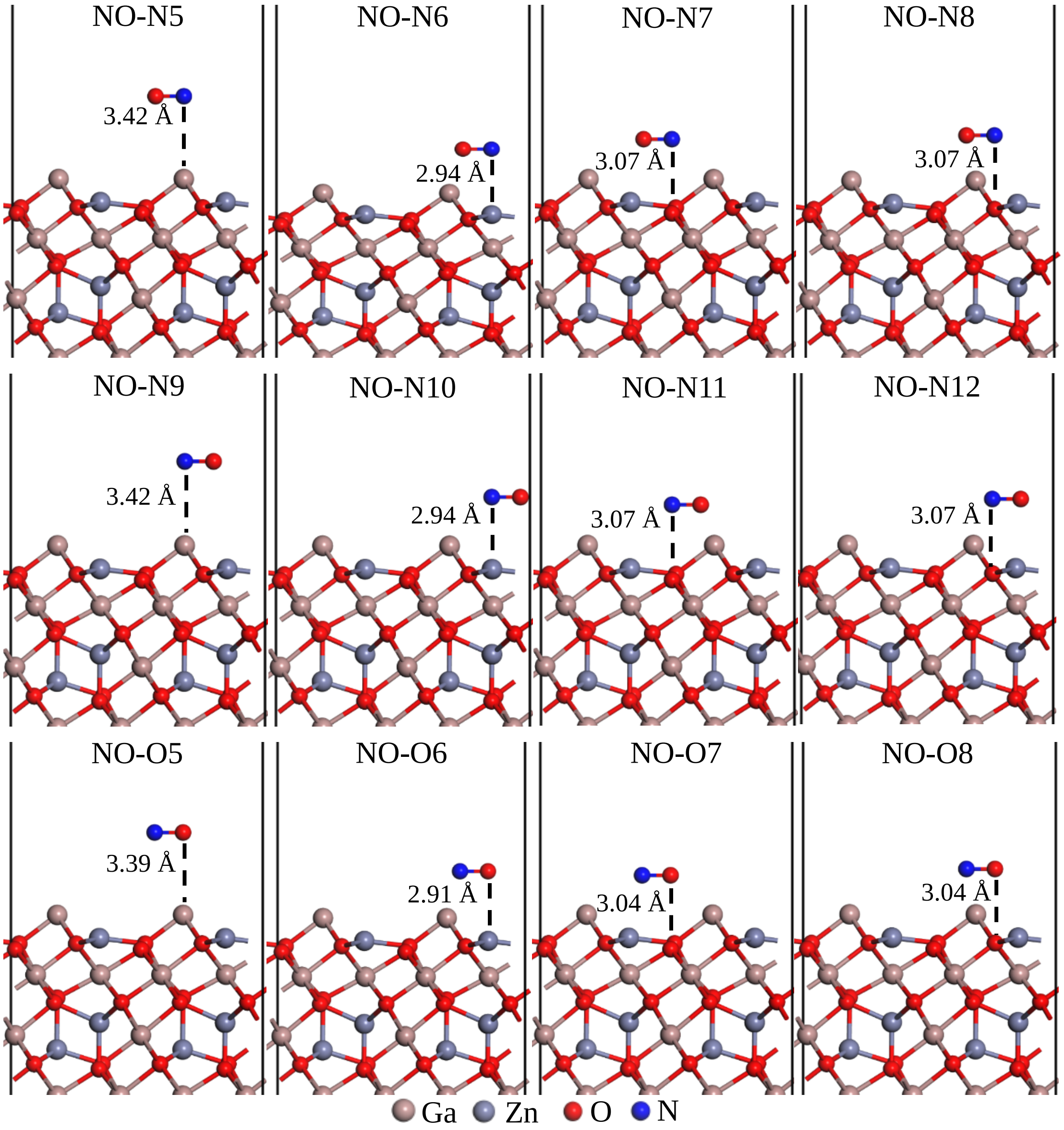

Figure S2 Initial configurations of NO molecules horizontally adsorbed on the $ZnGa_2O_4$(111) surface prior to geometric optimization. Models NO-N5 to NO-N8 represent NO molecules oriented horizontally with the nitrogen atom directed to the right, placed at the preferred adsorption sites on the $ZnGa_2O_4$(111) surface. Models NO-N9 to NO-N12 represent NO molecules oriented horizontally with the nitrogen atom directed to the left. Models NO-O5 to NO-O8 represent NO molecules oriented horizontally with the oxygen atom directed to the right toward the preferred adsorption sites. Ga atoms are shown in brown, Zn atoms in gray, O atoms in red, and N atoms in dark blue.
